# (*R*,*S*)-2′-Amino-6′-methyl-2,5′,5′-trioxo-6′*H*-spiro­[indoline-3,4′-pyrano[3,2-*c*][2,1]benzo­thia­zine]-3′-carbo­nitrile di­methyl­formamide monosolvate

**DOI:** 10.1107/S1600536814013634

**Published:** 2014-06-18

**Authors:** Svitlana V. Shishkina, Igor V. Ukrainets, Lidiya A. Petrushova

**Affiliations:** a STC "Institute for Single Crystals", National Academy of Sciences of Ukraine, 60 Lenina Ave., Kharkiv 61001, Ukraine; b National University of Pharmacy, 4 Blyukhera St, Kharkiv 61168, Ukraine

## Abstract

The title solvate, C_20_H_14_N_4_O_4_S·C_3_H_7_NO, comprises a stereogenic centre but the centrosymmetric space group causes the presence of the racemate in the crystal. The spiro-joined fragments are almost orthogonal, with a dihedral angle of 86.8 (2)° between the mean planes of the pyrane ring and the dihydroindolone ring system. The atoms of the indolinone bicycle are coplanar, with an r.m.s. deviation of 0.005 Å. In the crystal, pairs of N—H⋯O hydrogen bonds link the mol­ecules into centrosymmetric dimers which are linked to the di­methyl­formamide solvent mol­ecules by further N—H⋯O hydrogen bonds. N—H⋯N hydrogen bonds link neighbouring dimers into [010] chains.

## Related literature   

A three-component condensation of 1-*R*-4-hy­droxy-2-oxo-1,2-di­hydro­quinolines, isatin and malono­nitrile gave satisfactory yield of 4,3′-spiro­[(6-*R*-2-amino-5-oxo-5,6-di­hydro-4*H*-pyrano[3,2-*c*]quinoline-3-carbo­nitrile)-2′-oxindoles], see: Ukrainets *et al.* (2009 ▶). For van der Waals radii, see: Zefirov (1997 ▶) and for puckering parameters, see: Zefirov *et al.* (1990 ▶). For mean bond lengths, see: Bürgi & Dunitz (1994 ▶).
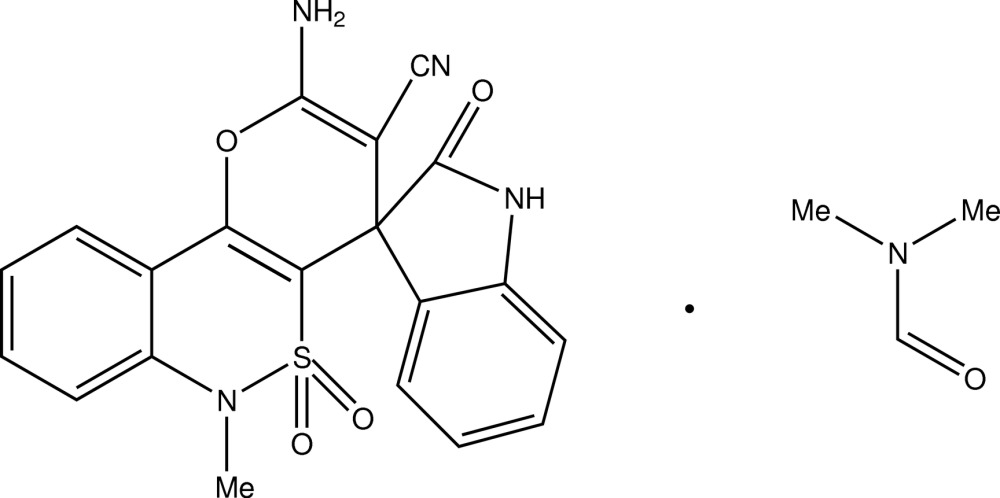


## Experimental   

### 

#### Crystal data  

C_20_H_14_N_4_O_4_S·C_3_H_7_NO
*M* *_r_* = 479.51
Orthorhombic, 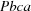

*a* = 17.2493 (12) Å
*b* = 9.6046 (5) Å
*c* = 27.7664 (17) Å
*V* = 4600.1 (5) Å^3^
*Z* = 8
Mo *K*α radiation
μ = 0.19 mm^−1^
*T* = 293 K
0.30 × 0.02 × 0.02 mm

#### Data collection  

Agilent Xcalibur"3 diffractometer
Absorption correction: multi-scan (*CrysAlis RED*; Agilent, 2011 ▶) *T*_min_ = 0.946, *T*_max_ = 0.996
30060 measured reflections
4049 independent reflections
2147 reflections with *I* > 2σ(*I*)
*R*_int_ = 0.034

#### Refinement  

*R*[*F* ^2^ > 2σ(*F* ^2^)] = 0.072
*wR*(*F* ^2^) = 0.194
*S* = 0.99
4049 reflections
323 parameters
H atoms treated by a mixture of independent and constrained refinement
Δρ_max_ = 0.44 e Å^−3^
Δρ_min_ = −0.27 e Å^−3^

### 

Data collection: *CrysAlis CCD* (Agilent, 2011 ▶); cell refinement: *CrysAlis RED* (Agilent, 2011 ▶); data reduction: *CrysAlis RED*; program(s) used to solve structure: *SHELXTL* (Sheldrick, 2008 ▶); program(s) used to refine structure: *SHELXTL*; molecular graphics: *XP* in *SHELXTL*; software used to prepare material for publication: *SHELXTL*.

## Supplementary Material

Crystal structure: contains datablock(s) global, I. DOI: 10.1107/S1600536814013634/kp2471sup1.cif

Structure factors: contains datablock(s) I. DOI: 10.1107/S1600536814013634/kp2471Isup2.hkl

CCDC reference: 1007876

Additional supporting information:  crystallographic information; 3D view; checkCIF report

## Figures and Tables

**Table 1:** Hydrogen-bond geometry (Å, °)

*D*—H⋯*A*	*D*—H	H⋯*A*	*D*⋯*A*	*D*—H⋯*A*
N1—H1*N*⋯O5^i^	0.82 (5)	2.01 (5)	2.797 (7)	160 (5)
N3—H3*NA*⋯O1^ii^	0.86 (5)	2.22 (5)	3.051 (6)	162 (4)
N3—H3*NB*⋯N4^iii^	0.83 (4)	2.37 (4)	3.159 (6)	160 (4)

Symmetry codes: (i) 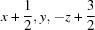; (ii) 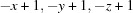; (iii) 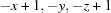.

**Fig. 1.**
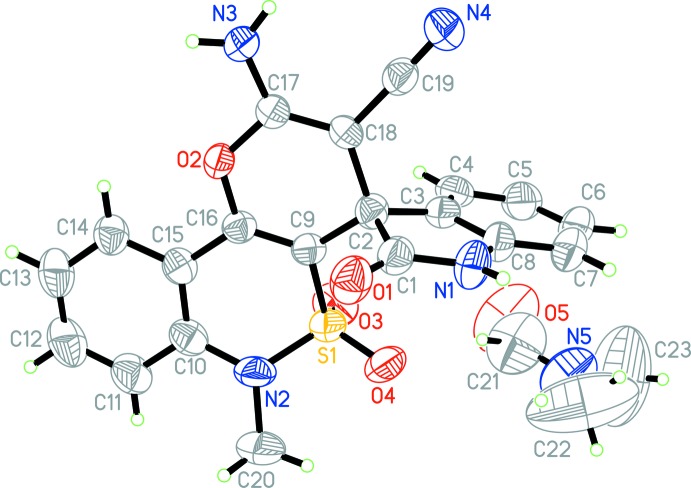
The title compound with atomic membering. All atoms are shown with displacement ellipsoids drawn at the 50% probability level.

**Fig. 2.**
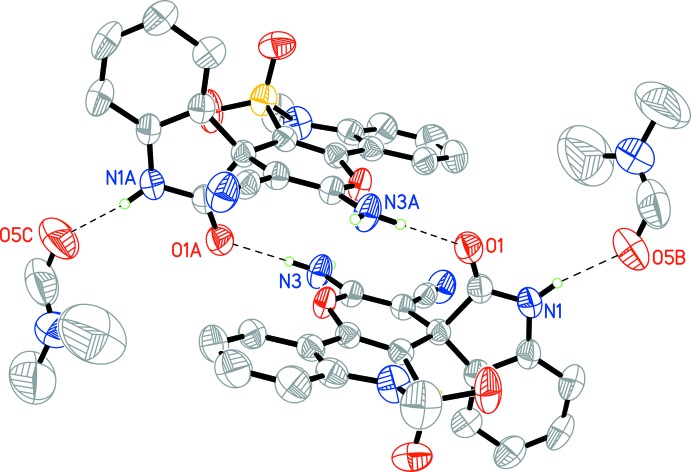
Hydrogen bonded, centrosymmetric dimers with dimethyl foramid solvate connected by hydrogen bonds.The hydrogen bonds are shown by dashed lines.

## supplementary crystallographic information

### S1. Comment

A three-component condensation of 1-*R*-4-hydroxy-2-oxo-1,2-dihydro- quinolines, isatin, and malononitrile gave a satisfactory yields of 4,3'- spiro[(6-*R*-2-amino-5-oxo-5,6-dihydro-4*H*-pyrano[3,2-*c*]quinoline-3- carbonitrile)-2'-oxindoles] (Ukrainets *et al.*, 2009). This is an interesting reaction takes place more easily with sulfo analogues of 1-*R*-4-hydroxy- 2-oxo-1,2-dihydroquinolines. For example, 1-methyl-4-oxo-3,4-dihydro-1*H*- 2λ^6^,1-benzothiazine-2,2-dione was already 15 minute after the start of the reaction forms the corresponding 2'-amino-6'-methyl-2-oxo-1,2-dihydro-6'H– spiro[indole-3,4'-pyrano[3,2-*c*][2,1]benzothiazine]3'-carbonitrile-5,5'- dioxide (I), which was isolated as a solvate of DMF. The spiro-joined tricyclic and bicyclic fragments of I are turned relatively to each other in such way that the dihedral angle between mean planes of the pyrane ring and dihydroindolone bicycle is 86.8°. At that the C1—C2 bond is elongated as compared with its mean value (Bürgi & Dunitz, 1994) 1.511 Å. The bicyclic fragment is slightly non-planar, the five-membered heterocycle adopts an envelope conformation with deviation of the C1 atom by 0.14 Å. In addition the dihedral angle between planar N1—C1(=O1)—C2 fragment and aromatic ring is 11.5 °. The pyrane ring adopts a boat conformation (the puckering parameters (Zefirov *et al.*, 1990) are: S=0.24, Θ=73.0°, Ψ=9.1°). Deviations of the O2 and C2 atoms from the mean plane of the remaining atoms of this ring are 0.10 Å and 0.20 Å, respectivey. The benzothiazine ring adopts a twist-boat conformation (the puckering parameters are S=0.57, Θ=47.2°, Ψ=25.9°). Deviations of the S1 and C9 atoms from the mean plane of the remaining atoms of this ring are -0.76 Å and -0.23 Å, respectivey. The steric repulsion between methyl group and atoms of the C10···C15 ring (shortened intramolecular contacts are: H20*b*···C11 2.71 Å, H11···C20 2.50 Å as compared with van der Waals radii sum ((Zefirov, 1997) 2.87 Å and H11···H20*b* 2.26 Å (2.34 Å)) results in elongation of the N2—C10 bond up to 1.415 (6) Å as compared with its mean value 1.371 Å. In the crystal the molecules form the centrosymmetric dimers (Fig. 2) by N3—H3Na···O1' (1 - *x*, 1 - *y*, 1 - *z*) intermolecular hydrogen bonds (Table 1). Each monomer of such dimer is bonded with DMF solvate molecule by the N1—H···O5' (0.5 + *x*, *y*, 1.5 - *z*) hydrogen bond. N3—H3Nb···N4' (1 - *x*, -*y*, 1 - *z*) hydrogen bond is observed between neighboring dimers (Table 1).

### S2. Experimental

A mixture of 1-methyl-4-oxo-3,4-dihydro-1*H*-2λ^6^,1-benzothiazine-2,2- dione (2.11 g, 0.01 mol), isatin (1.47 g, 0.01 mol), malononitrile (0.66 g, 0.01 mol), and triethylamine (1.4 ml, 0.01 mol) in methanol (20 ml) was refluxed for 15 min, and cooled. The precipitated were off, washed with methanol, and crystallized from DMF-H_2_O (1:1). Prepared 3.21 g (67%) solvate crystals of the pyranobenzothiazine with DMF; mp 437-439 K (decomp.).

### S3. Refinement

All hydrogen atoms were located from electron density difference maps and were refined in the riding motion approximation with *U*_iso_ constrained to be 1.5 times *U*_eq_ of the carrier atom for the methyl group and 1.2 times *U*_eq_ of the carrier atom for the other atoms. Hydrogen atoms of the aminogroups are refined using isotropic approximation.

### Crystal data

**Table:**

C_20_H_14_N_4_O_4_S·C_3_H_7_NO	*D*_x_ = 1.385 Mg m^−^^3^
*M**_r_* = 479.51	Melting point = 437–439 K
Orthorhombic, *P**b**c**a*	Mo *K*α radiation, λ = 0.71073 Å
Hall symbol: -P 2ac 2ab	Cell parameters from 2361 reflections
*a* = 17.2493 (12) Å	θ = 3.2–21.6°
*b* = 9.6046 (5) Å	µ = 0.19 mm^−^^1^
*c* = 27.7664 (17) Å	*T* = 293 K
*V* = 4600.1 (5) Å^3^	Stick, colourless
*Z* = 8	0.30 × 0.02 × 0.02 mm
*F*(000) = 2000	

### Data collection

**Table:**

Agilent Xcalibur"3 diffractometer	4049 independent reflections
Radiation source: Enhance (Mo) X-ray Source	2147 reflections with *I* > 2σ(*I*)
Graphite monochromator	*R*_int_ = 0.034
Detector resolution: 16.1827 pixels mm^-1^	θ_max_ = 25.0°, θ_min_ = 2.8°
ω scans	*h* = −20→17
Absorption correction: multi-scan (*CrysAlis RED*; Agilent, 2011)	*k* = −11→11
*T*_min_ = 0.946, *T*_max_ = 0.996	*l* = −33→33
30060 measured reflections	

### Refinement

**Table:**

Refinement on *F*^2^	Primary atom site location: structure-invariant direct methods
Least-squares matrix: full	Secondary atom site location: difference Fourier map
*R*[*F*^2^ > 2σ(*F*^2^)] = 0.072	Hydrogen site location: difference Fourier map
*wR*(*F*^2^) = 0.194	H atoms treated by a mixture of independent and constrained refinement
*S* = 0.99	*w* = 1/[σ^2^(*F*_o_^2^) + (0.0901*P*)^2^] where *P* = (*F*_o_^2^ + 2*F*_c_^2^)/3
4049 reflections	(Δ/σ)_max_ < 0.001
323 parameters	Δρ_max_ = 0.44 e Å^−^^3^
0 restraints	Δρ_min_ = −0.27 e Å^−^^3^

### Special details

**Table:**

Experimental. Absorption correction: CrysAlis RED, Agilent Technologies, Version 1.171.36.24 (release 03-12-2012 CrysAlis171 .NET) (compiled Dec 3 2012,18:21:49) Empirical absorption correction using spherical harmonics, implemented in SCALE3 ABSPACK scaling algorithm.
Geometry. All e.s.d.'s (except the e.s.d. in the dihedral angle between two l.s. planes) are estimated using the full covariance matrix. The cell e.s.d.'s are taken into account individually in the estimation of e.s.d.'s in distances, angles and torsion angles; correlations between e.s.d.'s in cell parameters are only used when they are defined by crystal symmetry. An approximate (isotropic) treatment of cell e.s.d.'s is used for estimating e.s.d.'s involving l.s. planes.
Refinement. Refinement of *F*^2^ against ALL reflections. The weighted *R*-factor *wR* and goodness of fit *S* are based on *F*^2^, conventional *R*-factors *R* are based on *F*, with *F* set to zero for negative *F*^2^. The threshold expression of *F*^2^ > σ(*F*^2^) is used only for calculating *R*-factors(gt) *etc*. and is not relevant to the choice of reflections for refinement. *R*-factors based on *F*^2^ are statistically about twice as large as those based on *F*, and *R*- factors based on ALL data will be even larger.

### Fractional atomic coordinates and isotropic or equivalent isotropic displacement parameters (Å^2^)

**Table:**

	*x*	*y*	*z*	*U*_iso_*/*U*_eq_	
S1	0.37888 (7)	0.59466 (11)	0.64282 (4)	0.0534 (4)	
O1	0.58546 (17)	0.4943 (3)	0.59913 (12)	0.0611 (9)	
O2	0.41460 (16)	0.4674 (3)	0.51036 (9)	0.0464 (7)	
O3	0.30029 (19)	0.5566 (3)	0.65291 (13)	0.0712 (10)	
O4	0.4331 (2)	0.5859 (3)	0.68127 (12)	0.0748 (10)	
O5	0.1982 (3)	0.3611 (7)	0.7857 (2)	0.157 (2)	
N1	0.5617 (3)	0.3404 (4)	0.66047 (15)	0.0579 (11)	
H1N	0.605 (3)	0.330 (5)	0.6722 (19)	0.073 (18)*	
N2	0.3820 (2)	0.7522 (3)	0.62123 (14)	0.0569 (10)	
N3	0.4424 (2)	0.2711 (5)	0.47288 (15)	0.0554 (11)	
H3NB	0.457 (2)	0.189 (4)	0.4712 (15)	0.048 (14)*	
H3NA	0.434 (2)	0.322 (4)	0.4479 (17)	0.057 (15)*	
N4	0.5120 (2)	0.0310 (4)	0.56205 (14)	0.0653 (12)	
N5	0.3135 (3)	0.3806 (7)	0.8231 (3)	0.1135 (19)	
C1	0.5437 (2)	0.4109 (4)	0.62037 (16)	0.0475 (11)	
C2	0.4597 (2)	0.3692 (4)	0.60525 (15)	0.0414 (10)	
C3	0.4343 (3)	0.2863 (4)	0.64885 (15)	0.0448 (11)	
C4	0.3633 (3)	0.2320 (4)	0.66055 (17)	0.0527 (12)	
H4	0.3212	0.2404	0.6398	0.063*	
C5	0.3562 (3)	0.1631 (4)	0.70495 (19)	0.0661 (14)	
H5	0.3082	0.1289	0.7146	0.079*	
C6	0.4193 (4)	0.1461 (5)	0.73416 (17)	0.0686 (14)	
H6	0.4136	0.0968	0.7628	0.082*	
C7	0.4917 (3)	0.2001 (5)	0.72248 (17)	0.0669 (14)	
H7	0.5344	0.1891	0.7426	0.080*	
C8	0.4966 (3)	0.2713 (4)	0.67924 (16)	0.0498 (11)	
C9	0.4128 (2)	0.4982 (4)	0.59449 (15)	0.0394 (10)	
C10	0.3533 (2)	0.7780 (4)	0.57434 (18)	0.0504 (11)	
C11	0.3224 (3)	0.9077 (4)	0.5625 (2)	0.0643 (14)	
H11	0.3168	0.9758	0.5861	0.077*	
C12	0.3004 (3)	0.9344 (5)	0.5162 (2)	0.0743 (16)	
H12	0.2806	1.0216	0.5084	0.089*	
C13	0.3070 (3)	0.8350 (5)	0.4807 (2)	0.0685 (14)	
H13	0.2911	0.8548	0.4495	0.082*	
C14	0.3368 (2)	0.7075 (4)	0.49171 (17)	0.0511 (11)	
H14	0.3412	0.6405	0.4677	0.061*	
C15	0.3609 (2)	0.6756 (4)	0.53878 (16)	0.0444 (11)	
C16	0.3960 (2)	0.5445 (4)	0.55022 (15)	0.0394 (10)	
C17	0.4408 (2)	0.3341 (4)	0.51557 (15)	0.0415 (10)	
C18	0.4615 (2)	0.2827 (4)	0.55921 (15)	0.0404 (10)	
C19	0.4899 (2)	0.1437 (4)	0.56126 (15)	0.0459 (11)	
C20	0.3838 (3)	0.8663 (4)	0.65669 (18)	0.0827 (18)	
H20C	0.3317	0.8908	0.6655	0.124*	
H20B	0.4091	0.9458	0.6429	0.124*	
H20A	0.4116	0.8367	0.6848	0.124*	
C21	0.2672 (5)	0.3931 (5)	0.7875 (2)	0.133 (3)	
H21	0.2881	0.4311	0.7595	0.159*	
C22	0.3923 (5)	0.4117 (12)	0.8231 (5)	0.251 (8)	
H22A	0.4055	0.4587	0.7938	0.376*	
H22B	0.4216	0.3270	0.8256	0.376*	
H22C	0.4040	0.4707	0.8501	0.376*	
C23	0.2835 (7)	0.3142 (14)	0.8647 (5)	0.259 (7)	
H23A	0.2449	0.2474	0.8553	0.389*	
H23B	0.2605	0.3825	0.8855	0.389*	
H23C	0.3248	0.2676	0.8814	0.389*	

### Atomic displacement parameters (Å^2^)

**Table:**

	*U*^11^	*U*^22^	*U*^33^	*U*^12^	*U*^13^	*U*^23^
S1	0.0699 (9)	0.0464 (7)	0.0440 (7)	0.0084 (6)	−0.0028 (6)	−0.0073 (5)
O1	0.054 (2)	0.072 (2)	0.057 (2)	−0.0097 (16)	−0.0099 (17)	0.0087 (17)
O2	0.065 (2)	0.0423 (15)	0.0317 (16)	0.0125 (13)	−0.0046 (14)	0.0015 (13)
O3	0.065 (2)	0.071 (2)	0.077 (3)	0.0050 (17)	0.0236 (19)	−0.0025 (17)
O4	0.102 (3)	0.066 (2)	0.056 (2)	0.0198 (18)	−0.034 (2)	−0.0182 (17)
O5	0.067 (3)	0.285 (7)	0.118 (5)	0.007 (4)	0.030 (3)	−0.012 (4)
N1	0.048 (3)	0.073 (3)	0.053 (3)	0.004 (2)	−0.012 (2)	0.012 (2)
N2	0.070 (3)	0.041 (2)	0.060 (3)	0.0093 (18)	−0.009 (2)	−0.0175 (18)
N3	0.083 (3)	0.046 (2)	0.037 (3)	0.013 (2)	−0.006 (2)	−0.004 (2)
N4	0.086 (3)	0.051 (2)	0.059 (3)	0.017 (2)	−0.001 (2)	0.002 (2)
N5	0.074 (4)	0.165 (5)	0.102 (5)	−0.014 (4)	0.002 (4)	−0.012 (4)
C1	0.049 (3)	0.050 (3)	0.044 (3)	0.005 (2)	−0.005 (2)	−0.001 (2)
C2	0.041 (3)	0.043 (2)	0.040 (3)	0.0064 (18)	−0.002 (2)	0.0000 (18)
C3	0.053 (3)	0.040 (2)	0.041 (3)	0.005 (2)	−0.002 (2)	−0.0063 (19)
C4	0.066 (3)	0.038 (2)	0.054 (3)	−0.003 (2)	0.004 (3)	−0.007 (2)
C5	0.086 (4)	0.057 (3)	0.055 (3)	−0.009 (3)	0.007 (3)	0.001 (3)
C6	0.104 (4)	0.067 (3)	0.035 (3)	−0.007 (3)	0.001 (3)	0.008 (2)
C7	0.088 (4)	0.068 (3)	0.044 (3)	−0.002 (3)	−0.010 (3)	0.010 (2)
C8	0.055 (3)	0.051 (2)	0.043 (3)	0.006 (2)	−0.007 (2)	0.006 (2)
C9	0.039 (2)	0.039 (2)	0.040 (2)	0.0001 (18)	−0.002 (2)	−0.0038 (19)
C10	0.045 (3)	0.043 (2)	0.063 (3)	0.0048 (19)	0.001 (2)	−0.001 (2)
C11	0.069 (3)	0.049 (3)	0.074 (4)	0.017 (2)	0.002 (3)	−0.001 (3)
C12	0.075 (4)	0.058 (3)	0.090 (5)	0.028 (3)	0.007 (3)	0.016 (3)
C13	0.066 (3)	0.067 (3)	0.072 (4)	0.020 (3)	−0.008 (3)	0.020 (3)
C14	0.050 (3)	0.053 (3)	0.051 (3)	0.009 (2)	0.000 (2)	0.006 (2)
C15	0.039 (3)	0.043 (2)	0.050 (3)	0.0034 (18)	0.001 (2)	0.003 (2)
C16	0.038 (2)	0.040 (2)	0.040 (3)	0.0008 (17)	−0.002 (2)	−0.0032 (19)
C17	0.047 (3)	0.039 (2)	0.039 (3)	0.0045 (18)	−0.001 (2)	−0.001 (2)
C18	0.043 (2)	0.034 (2)	0.044 (3)	0.0069 (17)	0.000 (2)	−0.0010 (18)
C19	0.051 (3)	0.051 (3)	0.035 (3)	0.002 (2)	0.002 (2)	0.000 (2)
C20	0.115 (5)	0.053 (3)	0.081 (4)	0.005 (3)	−0.012 (3)	−0.025 (3)
C21	0.104 (7)	0.184 (8)	0.110 (7)	0.023 (6)	0.044 (6)	−0.010 (6)
C22	0.105 (7)	0.364 (17)	0.284 (16)	−0.088 (9)	0.044 (8)	−0.183 (13)
C23	0.178 (11)	0.392 (19)	0.207 (14)	0.006 (11)	0.028 (10)	0.131 (13)

### Geometric parameters (Å, º)

**Table:**

S1—O4	1.422 (3)	C2—C3	1.514 (6)
S1—O3	1.432 (3)	C2—C18	1.525 (6)
S1—N2	1.628 (4)	C3—C4	1.370 (6)
S1—C9	1.732 (4)	C3—C8	1.374 (6)
O1—C1	1.228 (5)	C4—C5	1.404 (6)
O2—C17	1.366 (4)	C5—C6	1.367 (7)
O2—C16	1.370 (5)	C6—C7	1.390 (7)
O5—C21	1.231 (8)	C7—C8	1.384 (6)
N1—C1	1.339 (6)	C9—C16	1.339 (5)
N1—C8	1.404 (6)	C10—C11	1.394 (6)
N2—C10	1.415 (6)	C10—C15	1.400 (6)
N2—C20	1.474 (5)	C11—C12	1.365 (7)
N3—C17	1.331 (5)	C12—C13	1.375 (7)
N4—C19	1.148 (5)	C13—C14	1.363 (6)
N5—C21	1.277 (8)	C14—C15	1.406 (6)
N5—C22	1.391 (9)	C15—C16	1.432 (5)
N5—C23	1.417 (11)	C17—C18	1.357 (6)
C1—C2	1.560 (6)	C18—C19	1.423 (6)
C2—C9	1.510 (5)		
			
O4—S1—O3	117.4 (2)	C8—C7—C6	116.2 (5)
O4—S1—N2	108.0 (2)	C3—C8—C7	122.5 (5)
O3—S1—N2	109.9 (2)	C3—C8—N1	110.3 (4)
O4—S1—C9	109.15 (19)	C7—C8—N1	127.2 (5)
O3—S1—C9	109.5 (2)	C16—C9—C2	124.8 (4)
N2—S1—C9	101.58 (19)	C16—C9—S1	117.4 (3)
C17—O2—C16	119.9 (3)	C2—C9—S1	117.8 (3)
C1—N1—C8	111.3 (4)	C11—C10—C15	119.9 (5)
C10—N2—C20	119.5 (4)	C11—C10—N2	120.5 (4)
C10—N2—S1	119.3 (3)	C15—C10—N2	119.5 (4)
C20—N2—S1	116.5 (3)	C12—C11—C10	119.7 (5)
C21—N5—C22	126.3 (9)	C11—C12—C13	121.4 (4)
C21—N5—C23	116.4 (7)	C14—C13—C12	119.7 (5)
C22—N5—C23	116.9 (10)	C13—C14—C15	121.0 (4)
O1—C1—N1	126.4 (4)	C10—C15—C14	118.3 (4)
O1—C1—C2	125.6 (4)	C10—C15—C16	120.1 (4)
N1—C1—C2	108.0 (4)	C14—C15—C16	121.5 (4)
C9—C2—C3	115.7 (3)	C9—C16—O2	120.8 (3)
C9—C2—C18	107.0 (3)	C9—C16—C15	126.0 (4)
C3—C2—C18	112.9 (3)	O2—C16—C15	113.3 (3)
C9—C2—C1	110.0 (3)	N3—C17—C18	128.6 (4)
C3—C2—C1	100.9 (3)	N3—C17—O2	109.8 (4)
C18—C2—C1	110.3 (3)	C18—C17—O2	121.5 (4)
C4—C3—C8	120.9 (4)	C17—C18—C19	117.9 (4)
C4—C3—C2	130.5 (4)	C17—C18—C2	123.0 (3)
C8—C3—C2	108.6 (4)	C19—C18—C2	119.0 (3)
C3—C4—C5	117.7 (5)	N4—C19—C18	178.5 (5)
C6—C5—C4	120.5 (5)	O5—C21—N5	127.8 (6)
C5—C6—C7	122.1 (5)		
			
O4—S1—N2—C10	160.2 (3)	O3—S1—C9—C2	−97.2 (3)
O3—S1—N2—C10	−70.5 (4)	N2—S1—C9—C2	146.6 (3)
C9—S1—N2—C10	45.4 (4)	C20—N2—C10—C11	−3.9 (6)
O4—S1—N2—C20	−44.3 (4)	S1—N2—C10—C11	150.8 (3)
O3—S1—N2—C20	85.0 (4)	C20—N2—C10—C15	171.6 (4)
C9—S1—N2—C20	−159.1 (3)	S1—N2—C10—C15	−33.6 (5)
C8—N1—C1—O1	170.6 (4)	C15—C10—C11—C12	−0.5 (7)
C8—N1—C1—C2	−9.1 (5)	N2—C10—C11—C12	175.0 (5)
O1—C1—C2—C9	−47.7 (5)	C10—C11—C12—C13	0.9 (8)
N1—C1—C2—C9	132.0 (4)	C11—C12—C13—C14	−0.7 (8)
O1—C1—C2—C3	−170.4 (4)	C12—C13—C14—C15	0.1 (7)
N1—C1—C2—C3	9.3 (4)	C11—C10—C15—C14	−0.1 (6)
O1—C1—C2—C18	70.0 (5)	N2—C10—C15—C14	−175.6 (4)
N1—C1—C2—C18	−110.3 (4)	C11—C10—C15—C16	177.1 (4)
C9—C2—C3—C4	54.1 (6)	N2—C10—C15—C16	1.5 (6)
C18—C2—C3—C4	−69.7 (5)	C13—C14—C15—C10	0.3 (7)
C1—C2—C3—C4	172.7 (4)	C13—C14—C15—C16	−176.8 (4)
C9—C2—C3—C8	−124.9 (4)	C2—C9—C16—O2	6.7 (6)
C18—C2—C3—C8	111.3 (4)	S1—C9—C16—O2	−174.0 (3)
C1—C2—C3—C8	−6.4 (4)	C2—C9—C16—C15	−170.9 (4)
C8—C3—C4—C5	1.2 (6)	S1—C9—C16—C15	8.4 (6)
C2—C3—C4—C5	−177.7 (4)	C17—O2—C16—C9	9.0 (5)
C3—C4—C5—C6	−2.9 (7)	C17—O2—C16—C15	−173.1 (3)
C4—C5—C6—C7	2.7 (7)	C10—C15—C16—C9	11.0 (6)
C5—C6—C7—C8	−0.6 (7)	C14—C15—C16—C9	−171.9 (4)
C4—C3—C8—C7	0.8 (6)	C10—C15—C16—O2	−166.7 (4)
C2—C3—C8—C7	179.9 (4)	C14—C15—C16—O2	10.3 (5)
C4—C3—C8—N1	−177.5 (4)	C16—O2—C17—N3	169.2 (4)
C2—C3—C8—N1	1.6 (5)	C16—O2—C17—C18	−11.4 (6)
C6—C7—C8—C3	−1.1 (7)	N3—C17—C18—C19	0.8 (7)
C6—C7—C8—N1	176.9 (4)	O2—C17—C18—C19	−178.6 (3)
C1—N1—C8—C3	5.0 (5)	N3—C17—C18—C2	177.5 (4)
C1—N1—C8—C7	−173.2 (4)	O2—C17—C18—C2	−1.9 (6)
C3—C2—C9—C16	−144.0 (4)	C9—C2—C18—C17	14.6 (5)
C18—C2—C9—C16	−17.2 (5)	C3—C2—C18—C17	143.0 (4)
C1—C2—C9—C16	102.6 (5)	C1—C2—C18—C17	−105.0 (4)
C3—C2—C9—S1	36.8 (4)	C9—C2—C18—C19	−168.8 (3)
C18—C2—C9—S1	163.6 (3)	C3—C2—C18—C19	−40.3 (5)
C1—C2—C9—S1	−76.7 (4)	C1—C2—C18—C19	71.7 (5)
O4—S1—C9—C16	−146.6 (3)	C17—C18—C19—N4	−25 (22)
O3—S1—C9—C16	83.5 (3)	C2—C18—C19—N4	158 (22)
N2—S1—C9—C16	−32.7 (4)	C22—N5—C21—O5	176.6 (8)
O4—S1—C9—C2	32.7 (3)	C23—N5—C21—O5	4.2 (12)

### Hydrogen-bond geometry (Å, º)

**Table:**

*D*—H···*A*	*D*—H	H···*A*	*D*···*A*	*D*—H···*A*
N1—H1*N*···O5^i^	0.82 (5)	2.01 (5)	2.797 (7)	160 (5)
N3—H3*NA*···O1^ii^	0.86 (5)	2.22 (5)	3.051 (6)	162 (4)
N3—H3*NB*···N4^iii^	0.83 (4)	2.37 (4)	3.159 (6)	160 (4)

Symmetry codes: (i) *x*+1/2, *y*, −*z*+3/2; (ii) −*x*+1, −*y*+1, −*z*+1; (iii) −*x*+1, −*y*, −*z*+1.
